# Evaluation of genome similarities using a wavelet-domain approach

**DOI:** 10.1590/0037-8682-0470-2019

**Published:** 2020-05-18

**Authors:** Leila Maria Ferreira, Thelma Sáfadi, Juliano Lino Ferreira

**Affiliations:** 1 Universidade Federal de Lavras, Programa de Pós-Graduação Stricto Sensu em Estatística e Experimentação Agropecuária, Lavras, MG, Brasil.; 2 Universidade Federal de Lavras, Departamento de Estatística, Lavras, MG, Brasil.; 3 Empresa Brasileira de Pesquisa Agropecuária - Embrapa Pecuária Sul, Bagé, RS, Brasil.

**Keywords:** GC content, Hurst exponent, Mycobacterium tuberculosis, Discrete non-decimated wavelet transform, Grouping

## Abstract

**INTRODUCTION:**

Tuberculosis is listed among the top 10 causes of deaths worldwide. The resistant strains causing this disease have been considered to be responsible for public health emergencies and health security threats. As stated by the World Health Organization (WHO), around 558,000 different cases coupled with resistance to rifampicin (the most operative first-line drug) have been estimated to date. Therefore, in order to detect the resistant strains using the genomes of *Mycobacterium tuberculosis* (MTB), we propose a new methodology for the analysis of genomic similarities that associate the different levels of decomposition of the genome (discrete non-decimated wavelet transform) and the Hurst exponent.

**METHODS:**

The signals corresponding to the ten analyzed sequences were obtained by assessing GC content, and then these signals were decomposed using the discrete non-decimated wavelet transform along with the Daubechies wavelet with four null moments at five levels of decomposition. The Hurst exponent was calculated at each decomposition level using five different methods. The cluster analysis was performed using the results obtained for the Hurst exponent.

**RESULTS:**

The aggregated variance, differenced aggregated variance, and aggregated absolute value methods presented the formation of three groups, whereas the Peng and R/S methods presented the formation of two groups. The aggregated variance method exhibited the best results with respect to the group formation between similar strains.

**CONCLUSION:**

The evaluation of Hurst exponent associated with discrete non-decimated wavelet transform can be used as a measure of similarity between genome sequences, thus leading to a refinement in the analysis.

## INTRODUCTION

The genus Mycobacterium encompasses a broad set of gram-positive, acid-fast, rod-shaped microorganisms that are normally aerobic bacteria, and is the only member of the family *Mycobacteriaceae* within the order Actinomycetales. Like other narrowly related Actinomycetales, such as Nocardia and Corynebacterium, mycobacteria exhibit remarkably high GC content in their genomic DNA. They are capable of producing mycolic acid, which is a significant constituent of their cell wall. Mycobacterium tuberculosis (MTB) is considered as an active agent that causes tuberculosis (TB), which is a chronic infectious disease with growing incidence rate worldwide. This species is accountable for the highest morbidity in humans compared to other bacterial diseases. It infects around 1.7 billion individuals per year (≈33 % of the whole world inhabitants), and causes more than 3 million deaths per year on an average. This bacterium does not form a polysaccharide capsule and is an extremely slow-growing, aerobic, and obligatory parasite. The slow growth rate is attributed to the presence of a sturdy cell wall that resists the intake of nutrients by the cell and inhibits the excretion of waste products outside of the cell. The specialized cell envelope of this organism resembles the modified cell wall of a gram-positive bacterium^1^.

Due to the rising concern regarding the growing rate of deaths due to TB, studies have being carried out in order to target the drug resistant strains. Since the launch of the Global Project on Anti-tuberculosis Drug Resistance Surveillance in 1994, data on drug resistance have been collected and scrutinized from 160 countries worldwide (82 % of the 194 WHO Member States), which collectively have the data for over more than 97 % of the TB cases worldwide. Among with this, it includes 90 countries that have uninterrupted surveillance systems established on routine diagnostic drug susceptibility testing (DST) of all the TB patients, and 70 countries that depend on the epidemiological surveys carried out using the representative samples of TB patients. Surveys that are conducted every five years denote the most widespread approach for studying the burden of drug resistance in the resource-limited settings. Among the drug resistant strains, the most concerning are the multidrug resistant (MDR) and Extensively drug resistant (XDR) strains^2^.

Recently, the procedure of wavelets has increasingly been used for the analysis of bacterial genomes, such as wavelet packet analysis of amino acid chain sequences in the proteins of mesophile and thermophile bacteria^3^, comparative genomics via wavelet analysis for closely related bacteria^4^, discovery functional genetic material expression patterns in the metabolic pathways of Escherichia coli using wavelets transforms^5^, wavelet analysis to rapidly determine the characteristic morphology of the spore coat of bacteria^6^, and the existence of wavelet symmetries in Archaea DNA^7^. In a previous study, the authors bearing in mind the sequences of the MTB genome showed that the clustering analysis using the energy (variance) obtained at each decomposition level employing the discrete non-decimated wavelet transform (NDWT) was essential to verify the similarity of the sequences^8^. In another study, the authors used the combination of the two methodologies, including NDWT and Elastic net, and applied them in the analysis of clustering of the same strains of the MTB genome^9^. In this proposal, through the visualization of the graphs obtained by using the Elastic net method at each decomposition level, it was possible to identify the groups of similar strains. The GC content assessment also corresponds to one of the forms of bacterial genome analysis^10^. As the genome is composed of nitrogenous bases to form the DNA or RNA molecules, the GC content analysis transforms these bases into percentage that represents the signal to be analyzed employing an accurate statistic. Theoretically, the wavelet transform is a technique of observing and thus represents a signal^11^. This signal is decomposed at various resolution levels, where each level brings a detail, which corresponds with the multiresolution analysis^12^. Mathematically, it is characterized by a function that oscillates in time or space. In principle, it has sliding windows that expand or compress to capture low and high frequency signals, respectively^13^. We considered the discrete non-decimated wavelet transform (NDWT), whose main attribute is that it can work with any size of signals/sequences^14^ ^,^ ^15^. Studies encompassing the Hurst exponent were initially established in the field of hydrology for the practical matter of determining optimum size determination of dam for the Nile river's volatile rain and drought conditions that had been observed over a long period. The term "Hurst exponent" or "Hurst coefficient" was coined by Harold Edwin Hurst^16^, who was the lead researcher in these studies. Thereafter, the use of the standard notation H for the coefficient was also related to his name^17^. Its applicability in bacterial genome analysis was later demonstrated in the many different studies^18^ ^-^ ^21^. In this study, we aimed to verify the grouping of the strains with similar MTB genomes through the interaction between the two techniques, including non-decimated wavelet transform and Hurst exponent, and by applying five methods for the estimation of the Hurst exponent at each level of signal decomposition.

## METHODS

The sequences were chosen according to a previously described method^22^. Briefly, at the first instance of the analysis, it was important to obtain the signal referring to the strains of the genome of MTB. For this, the GC content was estimated with a sliding window of 10,000 base pairs (bp)^22^.

The GC content was determined as the ratio of the entirety of bases G and C, under the sum of the bases A, G, C, and T, according to the Equation 1:

$$GCcontent=\frac{nG+nC}{nA+nG+nC+nT}\;,$$ (1)

where nA, nG, nC, and nT represents the number of nucleotide bases A, G, C, and T, respectively, in a particular nucleotide sequence.

In Table 1, we have provided the description of the 10 analyzed sequences that were acquired from the National Center for Biotechnology Information database^1^, along with their corresponding total GC content estimates.

**TABLE 1:** Description of the *Mycobacterium tuberculosis* genome derived from different strains.

Sequence	NCBI Access	Resistance type	Total Rate of GC content	Infraspecific name
number	number			
Seq1	CP002992.1	DS	0.6560	CTRI-2
Seq2	CP000717.1	DS	0.6562	F11
Seq3	CP001641.1	DS	0.6561	CCDC5079
Seq4	CP001642.1	DR	0.6559	CCDC5180
Seq5	CP001664.1	DR	0.6563	str. Haarlem
Seq6	CP001658.1	MDR	0.6561	KZN 1435
Seq7	CP001976.1	XDR	0.6561	KZN 605
Seq8	CP002884.1	DS	0.6561	CCDC5079
Seq9	AL123456.3	DS	0.6561	H37Rv
Seq10	CP000611.1	DS	0.6561	H37Ra

**DS:** drug susceptible; **DR:** drug resistant; **MDR:** multidrug resistant; **XDR:** extensively drug resistant.

Once we obtained the signals of every single sequence, these signals were subjected to the phase of decomposition through the discrete non-decimated wavelet transform (NDWT), whose description is provided below^23^.

Considering ϕ and ψ as scaling and wavelet functions respectively, we here represent a data vector y=(y_0,y_1, …, y_m-1) of size m as a function *f* in terms of shifts of the scaling function at some multiresolution level *J* such that J-1<log_2 m≤J, as

$$\text{f}\left(\text{x}\right)\text{=}\sum_{\text{k=0}}^{\text{m-1}}{\text{y}}_{\text{k}}{\text{ϕ}}_{\text{J,k}}\text{(x)}\text{,}$$

where ϕ_J,k(x)=2^J/2ϕ(2^J(x-k)). The data interpolating function *f* can be re-expressed according to the Equation 2:

$$f\left(x\right)=\sum_{k=0}^{m-1}c_{J_{0},k}\phi_{J_{0},k}\left(x\right)+\sum_{j=J_{0}}^{J-1}\sum_{k=0}^{m-1}d_{jk}2^{j/2}\psi \left(2^{j}\left(x-k\right)\right),$$ (2)

where

$${\text{ϕ}}_{{\text{J}}_{\text{0}}\text{,k}}\left(\text{x}\right)\text{=}{\text{2}}^{{\text{J}}_{\text{0}}\text{/2}}\text{ϕ}\left({\text{2}}^{{\text{J}}_{\text{0}}}\left(\text{x-k}\right)\right),$$

$${\text{ψ}}_{\text{jk}}\left(\text{x}\right)\text{=}{\text{2}}^{\text{j/2}}\text{ψ}\left({\text{2}}^{\text{j}}\left(\text{x-k}\right)\right),$$

$$\text{j=}{\text{J}}_{\text{0}}\text{, …, J-1;k=0, 1, …, m-1}.$$

The coefficients c_(J_0,k) and d_j,k, j=J_0, …, J-1; k=0, …, m-1, represents the NDWT vector y.

We studied the Daubechies wavelet with 4 null moments and 5 levels of details, and the coefficients of each level are represented by (d1, d2, d3, d4, d5), where d1 corresponds to the level with less details and d5 to the level with more details^8^. 

The Hurst exponent corresponds to the range (0, 1), wherein for 0.5<H<1, it is said that the process has long-range dependence, for H=0.5 it is uncorrelated, while for 0<H<0.5, the process has short-range dependence^24^ ^-^ ^26^. Another interpretation, for example, in accordance with virology details that H<0.5 represents that the virus is locally confined, H≈0.5 represents that the virus behaves randomly, whereas H>0.5 represents a directed movement^27^. For the estimation of the Hurst exponent five methods were used in this study that are detailed as follows:

### Aggregated Variance Method

According to a previous study, one remarkable property of long-term memory processes is that the variance of the sample mean converges to zero slower than the rate N^(-1), where N is the sample size^28^. Here we assumed that

$$Var\left({\overset{-}{X}}_{N}\right)\sim cN^{2H-2}.$$ (3)

for large N, where c>0 and X¯_N represents the sample mean. This approach suggests the following method for estimating H, where the series is divided into N/m blocks of size m, and in every single block the sample mean is calculated according to the Equation 4:

$${\overset{-}{X}}_{m}\left(k\right)=\frac{1}{m}\sum_{t=\left(k-1\right)m+1}^{km}X\left(i\right),\;\;\;k=1,\;2,\;\ldots ,\;N/m.$$ (4)

and the sample variance is calculated according to the Equation 5:

$$s^{2}\left(m\right)={\left(N/m-1\right)}^{-1}\sum_{k=1}^{N/m}{\left({\overset{-}{X}}_{m}\left(k\right)-{\overset{-}{X}}_{N}\right)}^{2},$$ (5)

where X¯_N denotes the overall mean. Upon plotting logs^2(m) versus log(m), it should yield points scattered along a straight line with slope equal to 2H-2.

### Differenced Aggregated Variance Method

This is a method for discovering long-range dependence despite the presence of nonstationarity^29^. It is a variance-type estimator acquired by taking the logarithm of the first-order difference of Equation 4, which is presented as Equation 6:

$$\log∆Var\left[{\overset{-}{X}}_{m}\left(k\right)\right]\sim \log\frac{d}{dm}Var{\overset{-}{X}}_{m}\left(k\right)+\log∆m.$$ (6)

On one hand,

$$\frac{d}{dm}Var\left[{\overset{-}{X}}_{m}\left(k\right)\right]\sim \left(2H-2\right)Cm^{2H-3}.$$ (7)

Since the m values are logarithmically spaced, we further represent it as

$$\text{∆log}\left(\text{m}\right)\text{=const}\text{; that is, log}\text{∆m=}\text{log}\text{m+}{\text{C}}_{\text{1}}\text{.}$$

Therefore,

$$\text{log∆Var}\left[{\overset{-}{\text{X}}}_{\text{m}}\left(\text{k}\right)\right]\text{=}\left(\text{2H-3}\right)\text{log}\text{m}\text{+}\text{log}\left(\text{2H-2}\right)\text{C+}\text{log}\text{m+}{\text{C}}_{\text{1}}\text{=}\left(\text{2H-2}\right)\text{log}\text{m+}{\text{C}}_{\text{2}}.$$ (8)

Thus, in a log-log plot we would expect to obtain a straight line with a slope equal to 2H-2.

### Aggregated Absolute Value Method

Considering the series defined in Equation 4, and by computing its *n-th* absolute moment^30^

$${AM}_{n}^{\left(m\right)}=\frac{1}{\left(N/m\right)}\sum_{k=1}^{\left(N/m\right)}{\left|{\overset{-}{X}}_{m}(k)-{\overset{-}{X}}_{N}\right|}^{n},$$ (9)

AM_n^(m) is found to be asymptotically proportional to m^n(H-1).

To find an estimate of H, we have to compute AM_n^(m) for different values of m, and then generate a log-log plot against m. Here, we would expect that the points should be scattered along a straight line with slope n(H-1).

### Peng Method

According to^31^ a previous study, this method constitutes of the following steps: compute the partial sum within each block of size m according to the Equation 10:

$${Y\left(k\right)}^{m}=\sum_{t=\left(k-1\right)m+1}^{km}X\left(t\right),\;\;\;k=1,\;2,\;\ldots ,\;\left(N/m\right);$$ (10)

fit a regression line y=a+bk; compute the variance of the residual

$${\text{s}}_{\text{r}}^{\text{(m)}}\text{=}\frac{\text{1}}{\text{m}}\sum_{\text{k=1}}^{\text{N/m}}{\left({\text{Y}\left(\text{k}\right)}^{\text{m}}\text{-a-bk}\right)}^{\text{2}};$$

Plot logs_r^(m) vs log m; and then the slope should be equal to 2H.

### R/S Method

According to this method, R/S^32^can be estimated as follows: 

First, consider X_1,X_2, …, X_N as the observations and let

$${\text{Y}}_{\text{t}}\text{=}\sum_{\text{i=1}}^{\text{t}}{\text{X}}_{\text{i}}$$

be the partial sums. 

Define the adjusted range

$$R\left(t,k\right)=\underset{0\leq i\leq k}{\max}\left[Y_{t+i}-Y_{t}-\frac{i}{k}\left(Y_{t+k}-Y_{t}\right)\right]-\underset{0\leq i\leq k}{\min}\left[Y_{t+i}-Y_{t}-\frac{i}{k}\left(Y_{t+k}-Y_{t}\right)\right].$$ (11)

Consider

$$S\left(t,k\right)=\sqrt{k^{-1}\sum_{i=t+1}^{t+k}{\left(X_{i}-{\overset{-}{X}}_{t,k}\right)}^{2}}\;,$$ (12)

where

$${\overset{-}{\text{X}}}_{\text{t,k}}\text{=}{\text{k}}^{\text{-1}}\sum_{\text{i=t+1}}^{\text{t+k}}{\text{X}}_{\text{i}}.$$

The standardized ratio

$$Q\left(t,k\right)=\frac{R\left(t,k\right)}{S\left(t,k\right)}\;.$$ (13)

is known as rescaled adjusted range or R/S - statistic.

For the River Nile data, Hurst (1951) observed that, for large k,

$$\log E\left(R/S\right)\approx a+H\log k$$ (14)

with H>1/2.

Based on Hurst’s empirical findings, we can perform the following steps: divide the series into k block of size N/k; compute the R/S statistics Q(t_i,k), as defined in Equation (13), with starting values t_i=iN/k+1 for all possible k such that t_i+k<N; plot its logarithm against the logarithm of k; and then the estimated slope of the regression plot will be the estimate of H.

The values of Hurst exponent obtained at each level are considered in the cluster analysis. The clustering analysis was performed using each method with the distance of Mahalanobis in a hierarchical method with the average linkage. 

All the analyses and the generation of figures were carried out using the free software R (version 3.4.0)^33^. The packages used were seqinr, waveslim, fArma, and cluster^34^ ^-^ ^37^. The number of groups to be included in each method were estimated using the package NbClust^38^. (Supplmentary Data). 

## RESULTS

In this section, we have mainly presented in detail the analysis of the aggregated variance method.

In Table 2, we have presented the values calculated for the Hurst exponent at each decomposition level. It is important to note that at each level of decomposition, the value of the Hurst exponent is less than 0.5, thereby indicating short-range dependence.

**TABLE 2:** Hurst exponents obtained in the aggregated variance method.

Sequences	Levels				
	Level 1	Level 2	Level 3	Level 4	Level 5
Seq1_DS	-0.1764	0.0128	-0.1432	-0.0692	0.0723
Seq2_DS	-0.0707	0.0251	-0.2434	0.0858	0.0513
Seq3_DS	-0.1070	0.0170	-0.2113	0.0471	0.0705
Seq4_DR	-0.1303	-0.0515	-0.1438	0.0648	0.0499
Seq5_DR	-0.0362	-0.0401	-0.2597	0.0257	0.0771
Seq6_MDR	-0.0167	0.0233	-0.1412	0.0400	0.1977
Seq7_XDR	-0.0490	0.0176	-0.1443	0.0347	0.1979
Seq8_DS	-0.1711	0.0331	-0.2831	0.0765	0.0595
Seq9_DS	-0.1537	0.0068	-0.4009	0.0759	0.0604
Seq10_DS	-0.2241	-0.0554	-0.1840	0.0714	0.0506

**FIGURE 1:**
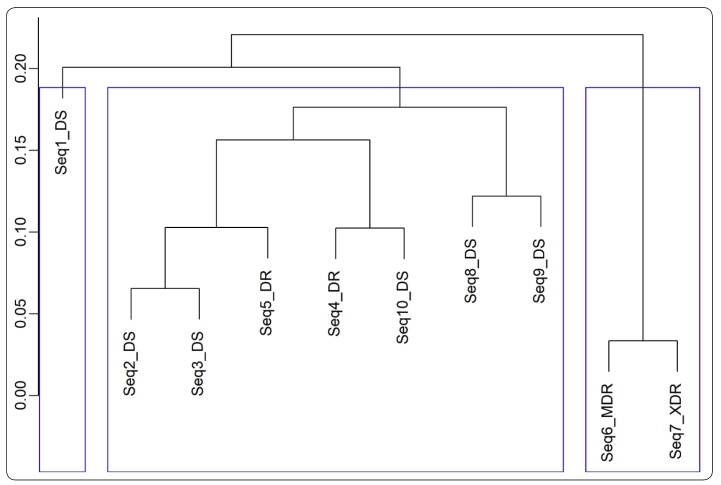
Clustering the sequences according to the aggregated variance method.

In Figure 1, the formation of three groups in accordance with the aggregated variance method is presented. The first group is formed only by the sequence Seq1_DS, a strain that was isolated from Russia affiliating to the AI family (consistent with the RFLP genotyping), and is susceptible to all the predicted drugs used in the treatment of tuberculosis. The sequences that appeared in the second group are as follows: Seq2_DS, a susceptible strain embodying majority of the part of patient’s diseased isolates that were recovered during an epidemic in the Western Cape of South Africa; Seq3_DS, a susceptible strain affiliated to the Beijing family that was sequenced for comparative genomic studies; Seq5_DR, a drug-resistant strain, exhibiting accelerated rate of transmission between humans especially under agglomeration conditions; Seq4_DR, a resistant strain isolated in 2004 from a patient with secondary pulmonary tuberculosis, and sequenced for comparative genomic studies; Seq10_DS, a virulent susceptible strain derived from its virulent parent strain H37Rv, which was isolated in 1905 and belongs to Edward R. Baldwin (19-year-old), a patient diagnosed with chronic pulmonary tuberculosis (this strain was acquired over an aging and dissociation procedure of an *in vitro* culture in the year 1935); Seq8_DS, a susceptible strain used for comparative genomic studies; and Seq9_DS, a susceptible strain derived from the original human lung H37Rv, and was isolated in 1934 (this strain has been broadly used all over the world in biomedical research. In contract to some clinical isolates, it retains total virulence in animals with tuberculosis and is susceptible to drugs and is approachable for genetic manipulation). The sequences that appeared in the third group include Seq6_MDR and Seq7_XDR, and both these sequences correspond to a particular patient from KwaZulu-Natal, South Africa. The results obtained for the Hurst exponent at each level were analyzed according to the following methods: differenced aggregated variance, aggregated absolute value, Peng, and R/S, and the details are presented in Table 3. 

**TABLE 3:** Hurst exponents obtained in the methods: differenced aggregated variance, aggregated absolute value, Peng, and R/S.

Differenced aggregated variance method					
Sequences	Levels				
	Level 1	Level 2	Level 3	Level 4	Level 5
Seq1_DS	0.0856	0.2538	0.1420	0.4270	0.8014
Seq2_DS	0.1829	0.3861	0.0152	0.5178	0.7536
Seq3_DS	0.2079	0.2289	0.0406	0.5227	0.7516
Seq4_DR	0.1106	0.1875	0.2269	0.5156	0.7400
Seq5_DR	0.2563	0.2904	0.0615	0.4320	0.7598
Seq6_MDR	0.1837	-0.0118	0.0230	0.4316	0.7683
Seq7_XDR	0.2818	-0.0551	0.0289	0.4454	0.8397
Seq8_DS	0.0153	0.0968	0.0447	0.5061	0.7775
Seq9_DS	-0.0902	0.1226	-0.0757	0.5057	0.8092
Seq10_DS	-0.1168	0.2491	0.0808	0.5249	0.8382
**Aggregated absolute value method**					
Seq1_DS	-0.0812	0.1258	-0.0419	0.0219	0.1731
Seq2_DS	0.0584	0.1269	-0.1355	0.1846	0.1430
Seq3_DS	-0.0061	0.1291	-0.0976	0.1442	0.1668
Seq4_DR	-0.0064	0.0583	-0.0196	0.1615	0.1403
Seq5_DR	0.0804	0.0738	-0.1317	0.1101	0.1702
Seq6_MDR	0.0781	0.1282	-0.0354	0.1448	0.2949
Seq7_XDR	0.0588	0.1265	-0.0382	0.1398	0.2947
Seq8_DS	-0.0432	0.1368	-0.1687	0.1731	0.1505
Seq9_DS	-0.0592	0.0999	-0.2610	0.1796	0.1560
Seq10_DS	-0.0900	0.0432	-0.0708	0.1713	0.1409
**Peng method**					
Seq1_DS	-0.0090	0.0088	0.2287	0.6945	1.1861
Seq2_DS	-0.0164	0.0114	0.2075	0.6817	1.1802
Seq3_DS	-0.0144	-0.0045	0.2109	0.6856	1.1813
Seq4_DR	-0.0124	0.0010	0.2165	0.6849	1.1950
Seq5_DR	-0.0149	0.0006	0.2049	0.6841	1.1862
Seq6_MDR	-0.0124	0.0027	0.2189	0.6986	1.2021
Seq7_XDR	-0.0111	0.0026	0.2202	0.6990	1.2016
Seq8_DS	-0.0121	0.0015	0.2081	0.6839	1.1846
Seq9_DS	-0.0126	-0.0059	0.1983	0.6811	1.1868
Seq10_DS	-0.0128	0.0078	0.1951	0.6813	1.1786
**R/S method**					
Seq1_DS	0.2208	0.2773	0.3641	0.6241	0.8847
Seq2_DS	0.1870	0.2087	0.3635	0.6892	0.8398
Seq3_DS	0.1756	0.2646	0.3427	0.6955	0.8417
Seq4_DR	0.1638	0.1951	0.3211	0.7044	0.8407
Seq5_DR	0.2018	0.2610	0.3736	0.6772	0.8609
Seq6_MDR	0.2329	0.2768	0.3811	0.6395	0.8985
Seq7_XDR	0.2234	0.2669	0.3785	0.6362	0.9009
Seq8_DS	0.1620	0.1987	0.3167	0.7042	0.8440
Seq9_DS	0.2055	0.2214	0.3207	0.6935	0.8420
Seq10_DS	0.2258	0.2530	0.3315	0.6953	0.8404

**FIGURE 2:**
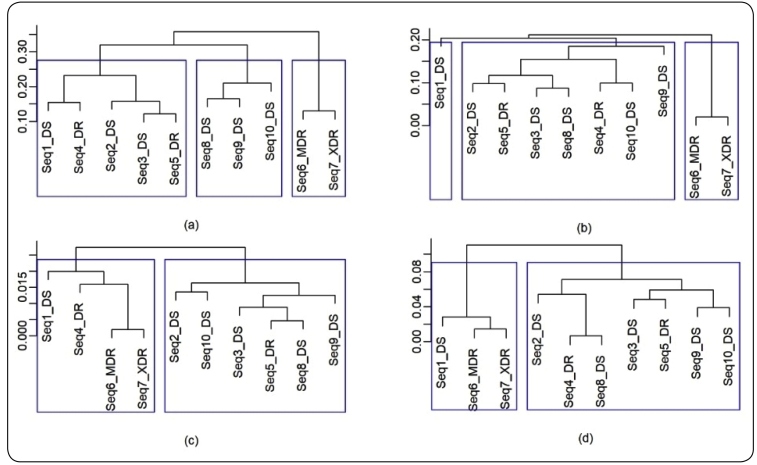
Clustering the sequences by using (a) the differenced aggregated variance method, (b) the aggregated absolute value method, (c) the Peng method, and (d) the R/S method

The results of the formation of groups according to the Differenced Aggregated Variance, Aggregated Absolute Value, Peng, and R/S methods are presented in the Figure 2a-d, respectively.

It is important to note that for aggregated variance and aggregated absolute value/moment methods, all the decomposition levels exhibited H less than 0.5, while the R/S, Peng, and differenced aggregated variance methods exhibited H less than 0.5 for the first three levels and more than 0.5 for the last two levels indicating long-range dependence. The negative values of H obtained in some methods were mainly because the estimated H was empirical, and this attributed to a negative or above 1 value of H^39^. The above 1 value of H obtained in the Peng Method was also reported in a previous study^40^.

The Aggregated Variance, Differenced Aggregated Variance, and Aggregated Absolute Value methods presented the formation of three groups, but the Peng and R/S methods presented the formation of two groups.

## DISCUSSION

In Figure 1, the sequence Seq1_DS appears to be isolated from the other two groups. However, in a previous study, the sequence Seq1_DS was found to be present in the same group as that of the sequences Seq6_MDR and Seq7_XDR. Moreover, upon plotting the last decomposition level (not showed here), the sequence Seq1_DS was found to exhibit completely different behavior than that of the sequences Seq6_MDR and Seq7_XDR. Therefore, the interaction between the discrete non-decimated wavelet transform and the Hurst exponent could effectively detect this difference.

Upon analyzing the formation of the second group in Figure 1, we noticed that the results of our study are in accordance with the results obtained in a previous study^9^. This is because in each level of decomposition, the group formation is very similar between the previous study and our methods.

The Aggregated Absolute Value method presented the most similar pattern of the formation of groups to the aggregated variance method; however the formation of their larger group with similar sequences, as represented in the Figure 2b, does not match with the results obtained in the previous studies^8^ ^,^ ^9^.

The differenced aggregated variance, Peng, and R/S methods, as presented in the Figures 2a, 2c and 2d, respectively, also do not present coherence in the formation of groups with similar sequences with the results obtained in the previous studies^8^ ^,^ ^9^. 

Among the five methods that were used for the estimation of the Hurst exponent, the results of the aggregated variance method for the formation of groups with similar sequences of the MTB genome were more closely related to the results obtained in the previous studies^8^ ^,^ ^9^. Even though each method presented different patterns of group formation, in all the methods the sequences Seq6_MDR and Seq7_XDR were found to occur in the same group, which represents the most resistant strains.

The proposed methodology applied for the analysis of clustering of the strains with MTB genome exhibited relevant results. Therefore, this methodology can be applied to any type of genome. The use of the discrete non-decimated wavelet transform allows the utilization of the entire genome sequence without taking into consideration the length as the power of two. Also, there is no loss of information.

When compared to other methods that were tested in this work, the aggregated variance method presented the best results with respect to the group formation for the similar strains. The results of this study indicate that the Hurst exponent associated with the discrete non-decimated wavelet transform may be used appropriately as a measure of similarity between the genome sequences. This may further help in obtaining refinement in the analysis and detecting details that remain unnoticed.
