# Supplementary material for: Evaluation of genome similarities using a wavelet-domain approach
Source: Rev Soc Bras Med Trop. 2020 May 18;53:e20190470. doi: 10.1590/0037-8682-0470-2019 (PMC7269520; doi:10.1590/0037-8682-0470-2019)
Supplement: Supplementary file 1 [file 1678-9849-rsbmt-53-e20190470-suppl1.pdf]

Codes of the analysis protocol

```
library(seqinr)
# working with the fasta files
dados <- read.fasta(file = "Sequence.fasta")
dadosseq <- dados[[1]]
slidingwindowplot <- function(windowsize, inputseq)
{
  starts <- seq(1, length(inputseq)-windowsize, by = windowsize)
  n <- length(starts)    # Find the length of the vector "starts"
  chunkGCs <- numeric(n) # Make a vector of the same length as vector "starts", but just
containing zeroes
  for (i in 1:n) {
    chunk <- inputseq[starts[i]:(starts[i]+windowsize-1)]
    chunkGC <- GC(chunk)
    print(chunkGC)
    chunkGCs[i] <- chunkGC
  }
plot(starts,chunkGCs,type="l",xlab="Sequence Length",ylab="GC Content")
solucao <- list(starts=starts,chunkGCs=chunkGCs)
return(solucao)
}
b_1 <- slidingwindowplot(10000, dadosseq)
b_1[[2]]
```

```
library(waveslim)

# signal decomposition using the discrete non-decimated wavelet transform

wds_1 <- modwt(b_1[[2]], wf="la8", n.levels=5, boundary = "periodic")

library(fArma)

# calculating the Hurst exponent for each level of decomposition

wds_1[[1]]     # Seq1_DS
wds_2[[1]]     #Seq2_DS
wds_3[[1]]     #Seq3_DS
wds_4[[1]]     #Seq4_DR
wds_5[[1]]     #Seq5_DR
wds_6[[1]]     #Seq6_MDR
wds_7[[1]]     #Seq7_XDR
wds_8[[1]]     #Seq8_DS
wds_9[[1]]     #Seq9_DS
wds_10[[1]]    #Seq10_DS


aggvarFit(wds_1[[1]])@hurst$H        #Aggregated Variance Method
diffvarFit(wds_1[[1]])@hurst$H       #Differenced Aggregated Variance Method
absvalFit(wds_1[[1]])@hurst$H        #Aggregated Absolute Value Method
pengFit(wds_1[[1]])@hurst$H          #Peng Method
rsFit(wds_1[[1]])@hurst$H            #R/S Method

library(NbClust)

# check the number of groups formed
```

```
Nb <- NbClust(x, distance="euclidean", min.nc=2, max.nc=6, method="complete",
index="all")            # x corresponds to the file with the Hurst exponent calculation
                          for each level of decomposition
library(cluster)
# groups dendrogram
sc=agnes(x,method="average",metric="mahalanobis",stand=FALSE)
dendro=as.dendrogram(as.hclust(sc))
plot(dendro)
pltree(sc,xlab="(a)",ylab="",main="",sub="")
groups <- cutree(as.hclust(sc), k=2)
rect.hclust(as.hclust(sc), k=2, border="blue")
```
